# Water Regime Evolution of Large Seasonal Lakes: Indicators for Characterization and an Application in Poyang Lake, China

**DOI:** 10.3390/ijerph15112598

**Published:** 2018-11-21

**Authors:** Feng Huang, Bo Yan, Xiao Zhang, Dayong Zhao, Lidan Guo, Yuankun Wang, Ziqiang Xia

**Affiliations:** 1 College of Hydrology and Water Resources, Hohai University, Nanjing 210098, China; dyzhao@hhu.edu.cn (D.Z.); zqxia@hhu.edu.cn (Z.X.); 2 Changjiang River Scientific Research Institute, Changjiang Water Resources Commission, Wuhan 430015, China; byanhhu@163.com; 3 Bureau of Hydrology, Changjiang Water Resources Commission, Wuhan 430012, China; zxhhu1991@163.com; 4 International River Research Centre, Hohai University, Nanjing 210098, China; ldguohhu@163.com; 5 School of Earth Sciences and Engineering, Nanjing University, Nanjing 210093, China; yuankunw@nju.edu.cn

**Keywords:** seasonal lakes, water regime, indicators, evolution, spatio-temporal variations, environmental problem, Poyang Lake

## Abstract

Impacted by ongoing climate change and anthropogenic activities, large seasonal lakes experience water regime evolution, which raises challenges for the management of water resources and environment. The water regime evolution refers to the spatial and temporal alterations in the hydrological features of lakes. Characterizing the lake water regime and its alteration may help policymakers design effective adaption strategies. Therefore, total 47 hydrological indicators were proposed, considering intra-annual fluctuations, flood and drought features, and rate and frequency of water level variations. Combined with Mann-Kendall algorithm and Sen’s slope, the indicators were applied in Poyang Lake, a typically large seasonal lake in China, as a case study. The results revealed temporal and spatial variations in different hydrological indicators. The most dramatic alteration was the water level decline in October and November over the entire study phase, especially over the past 30 years. This was an urgent environmental problem that Poyang Lake faced, partially caused by the increased hydraulic gradient between southern and northern lake. It could trigger the drought occurring earlier, prolong the drought duration, and impair the wetland ecosystem. Environmental water requirements of both Poyang Lake and Yangtze River were suggested for regional sustainable development. The application in Poyang Lake showed the practicability and reliability of the indicators, which are applicable in international seasonal lakes. The series of indicators can be used in whole or in part, determined by the ecohydrological characters of a specific lake and the research objectives.

## 1. Introduction

Large freshwater lakes; which supply water resources for human society, provide habitat for aquatic organisms [1,2], and support the adjacent wetland and terrestrial ecosystems [3,4]; play important roles in socio-economic development and environmental protection [5]. Large seasonal lakes, periodically experiencing obvious flood and drought seasons, are characterized by naturally seasonal fluctuations of lake water levels [6]. In an ecohydrological perspective, the lake water regime is essential to the ecological processes of relevant ecosystems [7,8,9,10,11,12,13,14]. For instance, fish may migrate from rivers into lakes for propagation and foraging during the flood season [15,16,17]. The shoal water region around the lake may be the spawning ground, the availability of which is determined by the characteristics of flood. The location and its range are affected by the flood water level and the period is influenced by the occurrence time and duration of the flood. In Tonle Sap Lake (Cambodia), where water level oscillations directly influence the connectivity to floodplain habitats for fish, the flood is an essential element supporting freshwater fish community structure and the fish diversity that underpins the food web [18,19]. The number of days inundated with flood may be a key factor determining the geographical distribution of *Oncomelania* snails in the vast floodplains of the Dongting Lake, China [20]. The low water level during the dry season, associated with the small water storage, may impact the water environmental carrying capacity [21]. On lake littoral zones, winter water level drawdowns can decrease taxonomic richness of macrophytes and benthic invertebrates, and affect fish assemblages via indirect pathways like decreased food resources [22]. Under the combined impact of climate change and anthropogenic activities, the natural hydrological regime of large seasonal lakes may be disrupted and eventually impair the dependent ecosystems [23]. Therefore, it is important to characterize the lake water regime, investigate the water regime evolution, and provide the environmental manager with the information of urgent issues and the corresponding advices.

For riverine ecosystems, indicators have been proposed to characterize the flow regime and indicate the hydrological alterations. The widely applied method is the Indicators of Hydrologic Alteration, which characterize magnitude, frequency, duration, timing and change rate of the flow regime [24,25]. But for large seasonal lakes, sparse efforts have been made to analyze the indicators, leaving a knowledge gap in depicting the lake water regime and investigating the urgent environmental problems. Inspired by the achievements in the river flow regime, the study aims to fill this knowledge gap. Furthermore, Poyang Lake, a typically seasonal lake in China, serves as a case study to apply the proposed indicators and testify their reliability.

Due to the socio-economic and ecological importance of Poyang Lake, its hydrological alterations have attracted worldwide attentions. Poyang Lake is freely connected with the mainstream of Yangtze River. It receives runoff from its local sub-basins and discharges into Yangtze River. Its water level fluctuations are heavily influenced by its local basin effect and the Yangtze River blocking effect, which controls the inflows and outflows of Poyang Lake, respectively [26]. In order to explore the causal factors influencing the river-lake system, the Ministry of Sciences and Technology launched a research project in 2012, the National Basic Research Program of China, which achieved impressive results in the evolution of river-lake interactions, the impacts of Three Gorges Reservoir (TGR), and the influences on lake hydrology, water quality, and ecosystem [27].

Climate change affects hydrological processes and natural ecosystems throughout the Yangtze River basin, thereby exacerbating the challenges of water resources management and ecological conservation. Upstream snow and ice reserves, which are important in sustaining seasonal water availability, are likely to be affected substantially by climate change [28]. The summer precipitation of the Yangtze River basin decreased during 2000–2008 in comparison to 1979–1999. The rainfall decrease was related to the weakened ascending motion and reduced water vapor content, which was mainly related to the weakened southwesterly moisture flux into the Yangtze River region [29]. The El Nino Southern Oscillation (ENSO) and the Pacific Decadal Oscillation (PDO) are two important climate oscillations that affect hydrological processes of Yangtze River. Water discharge tends to be higher during the La Nina-PDO cold phase and lower during the El Nino-PDO warm phase [30]. In the Poyang Lake basin, the ENSO and Indian Ocean Dipole are major climate indices that significantly correlate with magnitude and frequency of floods of the following year [31].

Besides climate change, another important factor is anthropogenic activities, especially reservoir construction. The impacts of the TGR on the lake water level have attracted great attention. The reservoir regulation changed the hydrological processes of Yangtze River and further altered the river-lake interactions. For example, the TGR would increase the flood risk due to its water release in late-May to early-June for the preparation of downstream summer flood control [32]. But during July to September, the TGR would mitigate flood risks, allowing more lake water to Yangtze River and resulting in the reduction of water storage in Poyang Lake [33]. During the impoundment of TGR, the drop-off of water level in Yangtze River increased the hydraulic slope between Poyang Lake and Yangtze River, speeded up the drainage of Poyang Lake and caused abnormally low levels [34]. The conjunction of extreme meteorological droughts in the Poyang Lake basin and the upper Yangtze River basin, coinciding with the TGR impoundment, was the main cause of the low water level in Poyang Lake [35]. The TGR impoundment was not responsible for the increased number of drought events; it might intensify the droughts [36]. Besides the TGR, the reservoirs in the Poyang Lake basin also affected the lake water level through runoff regulation. More than 10,000 reservoirs have been constructed in the Poyang Lake basin, including 26 large reservoirs [37]. Furthermore, the reservoirs affected the lake water level due to sedimentation interception, which interrupted sediment transport and resulted in channel erosion. The sand mining was another important factor affecting lake bed topography and lake water level. The enlarged outflow cross-section due to extensive sand mining was a major reason for water level decline in the northern Poyang Lake [38].

The previous studies have achieved great progresses in researches on hydrological processes in Poyang Lake, including river-lake interactions [39,40,41], flood and drought characteristics [42,43,44,45,46], the acting mechanisms of anthropogenic activities and climate changes in water level alterations [47,48,49,50,51,52]. Despite numerous previous studies, which usually focused on a specific hydrological feature or the impact of a specific factor analyzed by simulation, a thorough spatial and temporal analysis on water level fluctuations in Poyang Lake is still lacking, leaving a gap for understanding the evolution of lake water regime under the context of ongoing climate change and anthropogenic activities.

Hence, this study considered a complete hydrological regime in a seasonal lake, proposed indicators for characterizing the lake water regime, and applied them in Poyang Lake. The indicators may also be applicable in other international seasonal lakes. As a case study, using updated daily water level data during the past 63 years, this study investigated the water regime in Poyang Lake, including long/short-term variations and spatial patterns. Assessing the historical temporal and spatial variations of lake water regime is important for developing effective management strategies for local water resources and for mitigation of future droughts and floods. The results may also provide scientific and practical references for the conservation and restoration of lacustrine and wetland ecosystems. The effects of climate change and human activities might accumulate or counteract each other simultaneously, but attempts were not made in this study to distinguish them.

## 2. Study Area and Data

**Figure 1:**
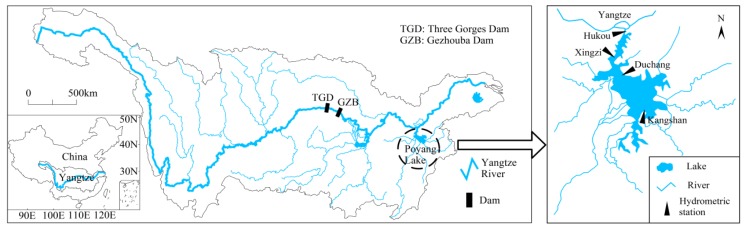
Sketch of Poyang Lake and locations of hydrometric stations.

Figure 1 depicts the study area and the locations of hydrometric stations in Poyang Lake. Yangtze River is one of the world’s great river systems, flowing approximately 6300 km through the central China. The Yangtze River basin drains a total area of 1.8 million km^2^ from the Qinghai-Tibet Plateau to the East China Sea. It encompasses a vast range of geographic and climatic zones; as a result, it is endowed with diverse and abundant natural resources. The Yangtze River basin has a large number of lakes, among which Poyang Lake is the largest freshwater lake in China and one of the most important international wetlands. Poyang Lake has very rich water and biological resources. It plays an important role in flood storage and regulation, water resources conservation, and maintenance of biological diversity and ecological security. It is situated within the East Asian Flyway, a migratory corridor for waterfowl. The Poyang Lake area is a famous bird sanctuary and its wetland is in the first batch of the Ramsar Convention List of Wetlands of International Importance.

Located at the junction of the middle and lower reaches of Yangtze River, Poyang Lake enters the Yangtze River’s right bank and empties into Yangtze River through a narrow river channel. The water level of Poyang Lake is influenced by the inflow water from the local five tributaries and the exchange of water with Yangtze River. Poyang Lake’s biodiversity is immense. The source of its great productivity is the seasonal variation in water level and the range of wetland habitats inundated. The area’s extensive wetland habitats would not exist without the annual flood. As a seasonal lake, Poyang Lake has a flood-drought cycle that significantly changes the water level every year. The yearly hydrological cycle can be divided into four phases: increasing water level, high water level, decreasing water level, and low water level. During the spring and summer flood season, it inundates a large area while in the winter it shrinks considerably, creating a large tract of marshland for wild migratory birds. The difference of water levels between the flood season and the dry season is within 8 m. The surface area is approximately 2370 km^2^ when the water level reaches its annual average value.

Four hydrometric stations, namely Kangshan, Duchang, Xingzi, and Hukou, monitor the water levels in Poyang Lake. Kangshan, Duchang, and Xingzi stations monitor the water level of southern, middle, and northern lake, respectively. Hukou station is located at the outlet of Poyang Lake, monitoring the water exchanges between Poyang Lake and Yangtze River. The water level data were daily recorded and the period spanned from 1953 to 2015. No data was missing from the records. Annual mean water level at Kangshan, Duchang, Xingzi, and Hukou station was 13.42 m, 12.04 m, 11.42 m, and 10.93 m, decreasing from south to north.

## 3. Methods

### 3.1. Water Level Indicators

Water level indicators contain a total of 47 hydrologic parameters (Table 1), which are categorized into 4 groups of hydrological features. Magnitude of monthly water level quantifies intra-annual variations in lake water regime. Maximum and minimum water levels restrict the range that the water level may fluctuate in a month. In Poyang Lake, periods during which the daily water level is above the 75th and below the 25th percentile are labeled flood and drought, respectively, because it is a traditional method to define the flood and drought in China. In other international lakes, the specific thresholds can be set according to the local traditions and the hydrological features of the lake. The indicators for flood/drought characterize their occurrence time, duration and severity. Starting time of flood/drought is the earliest Julian date in a year when the water level begins to be above/below the threshold of flood/drought. Duration of flood/drought is the total number of days which are labeled flood/drought. Mean water level of floods/droughts is the average value of water level during flood/drought. Extreme water level of floods is the highest water level during a flood, and extreme water level of droughts is the lowest water level during a drought. Rise rate and fall rate quantify how fast the lake water regime changes. Rise rate is an average value of all positive differences between consecutive daily water levels, while fall rate corresponds to the negative differences. Number of water level reversals, quantifying the frequency of water level changes, is the number of times that water level switches from rising period to falling period.

### 3.2. Trend Analysis

The water level indicators were calculated for each year, and the results were further analyzed by Mann-Kendall algorithm and Sen’s slope to investigate the temporal alterations at different time scales. For a time series of a water level indicator, the trend was analyzed at a long-term time scale of 6 decades and a short-term time scale of 3 decades, respectively. The long-term tendency was analyzed over the entire study phase. The short-term fluctuations were further investigated for different time intervals, i.e., 1956–1985, 1966–1995, 1976–2005 and 1986–2015.

To ascertain the presence of a statistically significant trend in the time series, Mann-Kendall (M-K) algorithm was employed. It is a non-parametric rank based procedure, which checks the null hypothesis of no trend versus the alternative hypothesis of the existence of an upward or downward trend. The statistic Z of the M-K algorithm follows the standard normal distribution with a mean of zero and variance of one. A positive or negative value of Z represents an increasing or decreasing trend, respectively. In a two-tailed test, the null hypothesis can be rejected at a significance level α if |Z| > Z_α/2_. The significance level α is set to be 0.05 in this study. Sen’s slope has been widely used for estimating the magnitude of trend in hydrological and meteorological time series. The approach involves calculating slopes for all the pairs of ordinal time points using the median of these slopes as an estimate of the overall slope. Detailed information on the M-K algorithm and Sen’s slope can be found in the published articles [53,54,55,56,57,58].

## 4. Results of Water Regime Evolution

### 4.1. Monthly Mean Water Levels

**Figure 2:**
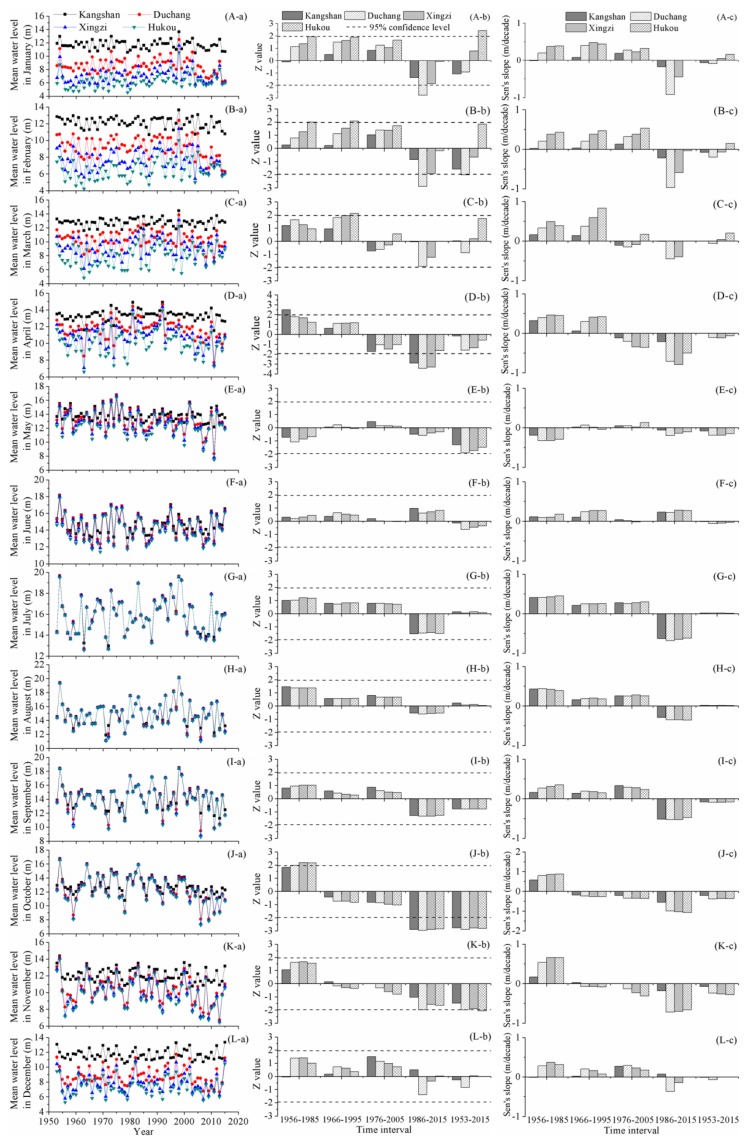
Change characteristics of monthly mean water level in Poyang Lake: (i) the sub-figures from the top to the bottom rows that are labeled (A–L) display monthly mean water level changes in January to December; and (ii) the sub-figures from the left to the right columns that are labeled (a–c) display annual values, Z values, and Sen’s slopes, respectively.

Figure 2 illustrates change characteristics of monthly mean water levels. In general, there were both spatial and temporal alterations. In January, there was a significant increase in average water level at Hukou station over 1953–2015. During 1986–2015, the average water levels at Kangshan, Duchang, and Xingzi stations declined, especially at Duchang station, where the decreasing tendency exceeded 95% confidence level and the decreasing rate reached approximately 1 m/decade. Average water level in February experienced similar change patterns with the average water level in January. The water level declined from south to north. Over the entire study phase, the average water level in February at Duchang station dropped significantly, while the water levels at Kangshan and Xingzi stations dropped slightly and the water level at Hukou station increased slightly. In the recent three decades, the average water level in February was dominated by a downward tendency. The water levels at Duchang and Xingzi stations declined significantly, with decreasing rates of about 1 m/decade and 0.6 m/decade, respectively.

In March, during 1956–1985 and 1966–1995, the water level tended to rise and the upward tendency exceeded 95% confidence level at Xingzi and Hukou stations over 1966–1995. Furthermore, the increasing rate at Hukou station during 1966–1995 was the largest, reaching 0.83 m/decade. Average water levels in April at Kangshan, Duchang, Xingzi, and Hukou stations experienced similar alterations, though the magnitudes of the changes were different. For the 4 sub-time intervals, the change patterns were different. The water level tended to increase during 1956–1985 and 1966–1995, whereas the downward trends were observed during 1976–2005 and 1986–2015. Over the past 30 years, the water level declined markedly and the trends were significant at Kangshan, Duchang, and Xingzi stations.

In October, for the time interval of 1956–1985, the monthly mean water level increased prominently; while since 1966, it started to decline and the decreasing trend became significant over the past three decades. The water levels at Kangshan, Duchang, Xingzi, and Hukou stations dropped simultaneously, with the significance exceeding 95% confidence level. The decreasing slope of the water level at Kangshan station was relatively small, equal to about 0.6 m/decade, while the water levels at Duchang, Xingzi, and Hukou stations decreased with the Sen’s slope of approximately 1 m/decade. Compared with the magnitudes of water level alterations in other months, there was the largest magnitude of water level reduction in October during the recent 30 years. Over the long-term period, the water level declined markedly, with the decreasing rate of about 0.2 m/decade at Kangshan station and the double decreasing rates at the rest 3 hydrometric stations. There were similar change patterns in average water levels of November and October. Over the past recent three decades, the decreasing rate increased to around 0.7 m/decade at Duchang, Xingzi, and Hukou stations and the downward tendency was significant at Duchang station.

In the remaining months, i.e., May-September and December, the monthly mean water levels fluctuated over 1953–2015, and some slight positive and negative alterations could be observed; but overall, there were not significant changes in different time intervals.

### 4.2. Monthly Maximum Water Levels

**Figure 3:**
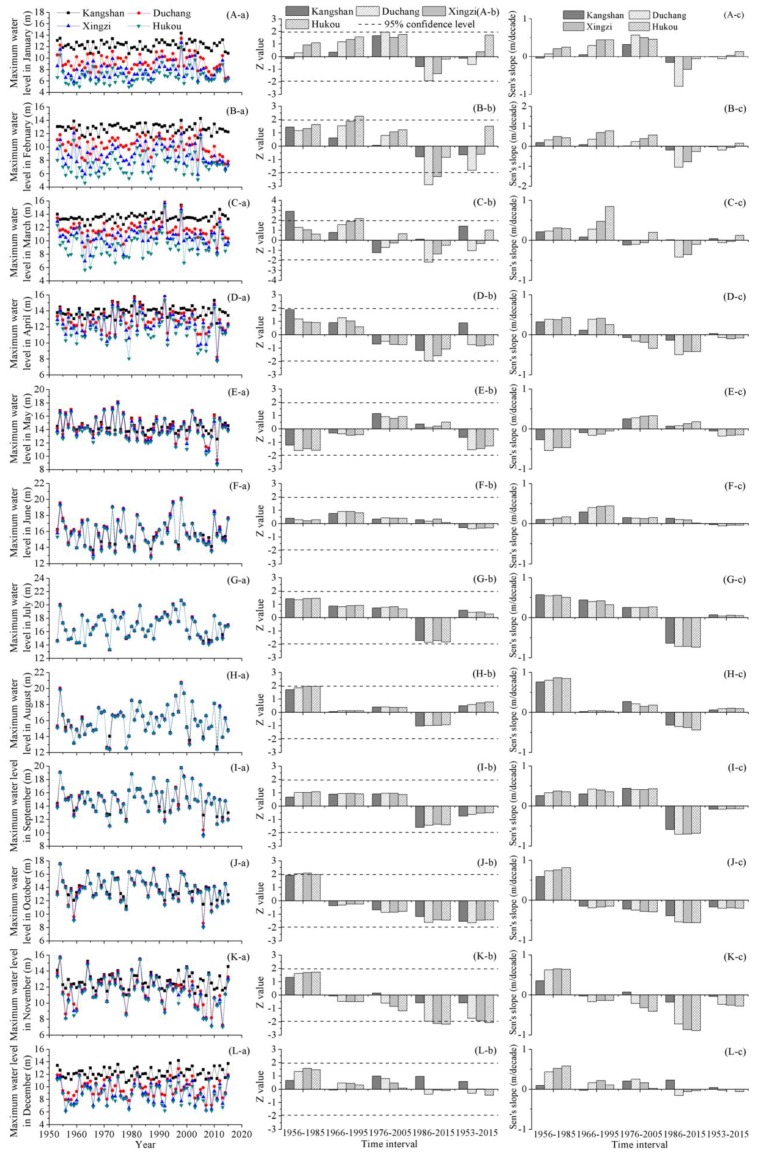
Change features of monthly maximum water level in Poyang Lake: (i) the sub-figures from the top to the bottom rows that are labeled (A–L) display monthly maximum water level changes in January to December; and (ii) the sub-figures from the left to the right columns that are labeled (a–c) display annual values, Z values, and Sen’s slopes, respectively.

Figure 3 displays yearly fluctuations, trends and change rates of monthly maximum water levels. In general, there were obvious temporal and spatial variations in the monthly maximum water levels. In January, during 1986–2015, the monthly maximum water level declined, and the significance of decreasing tendency of the water level at Duchang station exceeded 95% confidence level. Furthermore, there was the largest decreasing rate of the water level at Duchang station, almost 1 m/decade. The monthly maximum water level of February maintained an upward tendency until around 2000, with the increasing rates ranging from 0.01 to 0.76 m/decade. During 1966–1995, the magnitude of water level elevation followed an increasing gradient from Kangshan to Hukou station, and the upward trend in water level at Hukou station became significant. Over the past three decades, the monthly maximum water level in February declined markedly, particularly at Duchang and Xingzi stations. The decreasing rates of water levels at the four hydrometric stations ranged from 0.19 to 1.05 m/decade, with the largest value observed at Duchang station.

In March, some significant alterations were detected during the short-term time intervals. During 1956–1985 and 1966–1995, the change patterns were dominated by increasing tendencies: the increasing trends were significant at Kangshan station during 1956–1985 and at Hukou station during 1966–1995, respectively. From 1976 to 2005, the monthly maximum water level in March fluctuated without any significant trend, but from 1986 to 2015, it turned to decline and the downward trend was significant at Duchang station. The change features of monthly maximum water level in April were similar with that in March. During 1976–2005 and 1986–2015, the dominant tendency was a downward trend, which was significant at Duchang station from 1986 to 2015.

The change characteristics of monthly maximum water levers in October and November were similar. During 1956–1985, the monthly maximum water levels increased in varying degrees and the elevation in October passed the significance test. During the last five decades, downward trends were dominant and the decreasing rates increased gradually. From 1986 to 2015, the decline of monthly maximum water level in November was significant at Duchang, Xingzi, and Hukou stations and the magnitude of water level reduction was approximately 0.8 m/decade. At the long-term time scale, there were dominant tendencies of decrease in the monthly maximum water levels in October and November, and the water level decline in November was significant at Hukou station, with a Sen’s slope of about 0.3 m/decade.

Except for January to April and October to November, there was no significant trend in the monthly maximum water levels of the other months. During 1953–2015, the monthly maximum water levels in May-September and December remained relatively unchanged. At the time scale of 30 years, there were various indistinctive tendencies in the monthly maximum water levels in those months.

### 4.3. Monthly Minimum Water Levels

**Figure 4:**
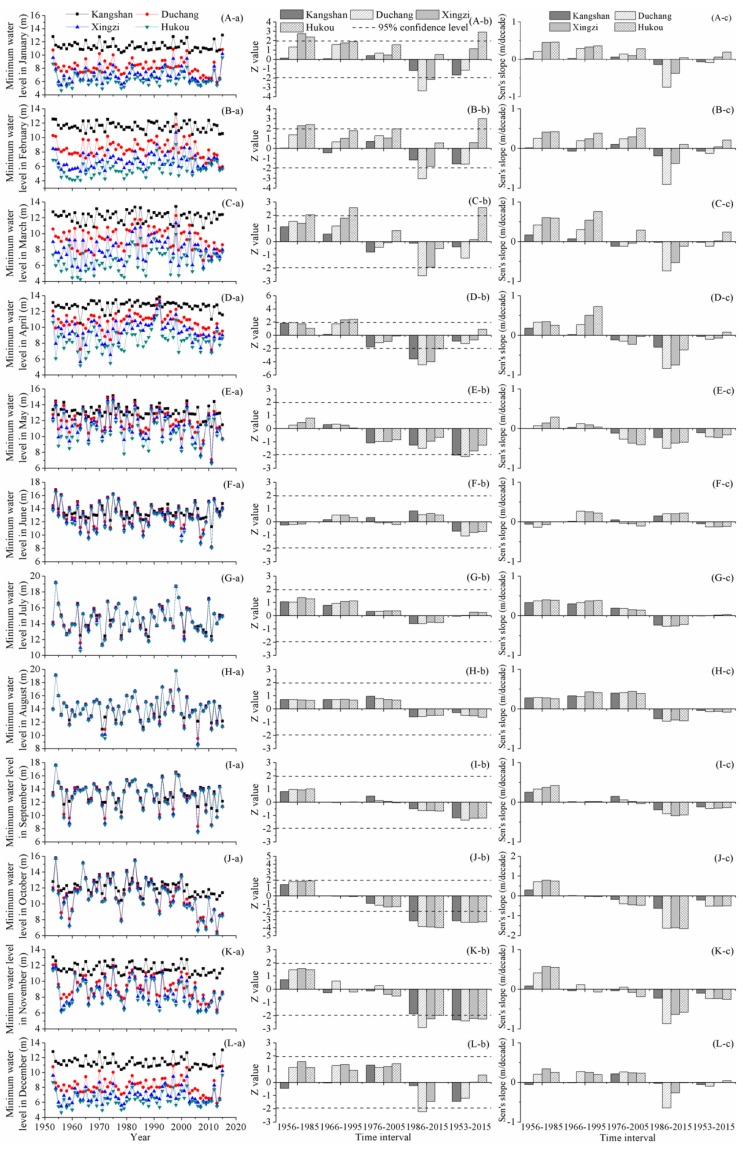
Variation features of monthly minimum water level in Poyang Lake: (i) the sub-figures from the top to the bottom rows that are labeled (A–L) display monthly minimum water level changes in January to December; and (ii) the sub-figures from the left to the right columns that are labeled (a–c) display annual values, Z values, and Sen’s slopes, respectively.

Temporal and spatial change characteristics of monthly minimum water levels are depicted in Figure 4. In January, at Duchang and Xingzi stations, the monthly minimum water levels maintained upward tendencies from 1950s to 2000s. Over the past three decades, the water levels experienced significant downward trends. At Hukou station, the monthly minimum water level in January increased consistently and the trend was significant over the past six decades. However, the increasing rate was small, only approximately 0.2 m/decade. Change patterns of the monthly minimum water level in February were similar to that in January. Hukou was the only station to observe a consistent increase in the monthly minimum water level. From 1953 to 2015, the water level significantly increased with a Sen’s slope of 0.2 m/decade. In recent 30 years, except for Hukou station, the monthly minimum water level declined at the other three hydrometric stations and the water level reduction was significant at Duchang station, with a decreasing slope of 0.9 m/decade.

In March, over 1956–1985 and 1966–1995, the monthly minimum water level increased to different extent, with the Sen’s slopes ranging from 0.07 to 0.76 m/decade. Additionally, the water level elevation followed an obvious south-to-north increasing gradient. During 1986 to 2015, the monthly minimum water level started to decrease. At Duchang station, the monthly minimum water level significantly decreased with a rate of 0.74 m/decade, being the largest magnitude of water level decline. The monthly minimum water level in April remained relatively unchanged over the entire study phase. Over the past three decades, there were prominent reductions in the monthly minimum water levels. The water level dropped the most dramatically at Duchang station, followed by Xingzi, Hukou, and Kangshan station. In May, at the long-term time scale, the monthly minimum water level was dominated by a downward tendency. Furthermore, the reductions of the monthly minimum water levels at Kangshan and Duchang stations were significant.

In October, the most distinct change in the monthly minimum water level was the significant decreasing trend over 1986–2015. The decreasing slope at Duchang, Xingzi, and Hukou stations was approximately 1.6 m/decade, ranking the largest amplitude of variation, compared with the change slopes in other months. The monthly minimum water level in November remained relatively unchanged during 1956–1985, 1966–1995 and 1976–2005. However, during 1986–2015, significant downward trends were detected and the decreasing slopes ranged from 0.23 to 0.87 m/decade at the four hydrometric stations. The most prominent water level reduction was monitored at Duchang station. From 1953 to 2015, the minimum water level in November declined evidently and the significance of trend was identified by the M-K algorithm at 95% confidence level. In December, only a significant decreasing trend was detected over the past 30 years at Duchang station, and the dropping slope reached 0.65 m/decade.

For the monthly minimum water levels in January–May and October–December, the M-K algorithm identified some significant alterations during the time intervals of short and long-term time scales. However, for the rest months, a common change characteristic was that no significant trend was detected in the monthly minimum water levels.

### 4.4. Flood Characteristics

**Figure 5:**
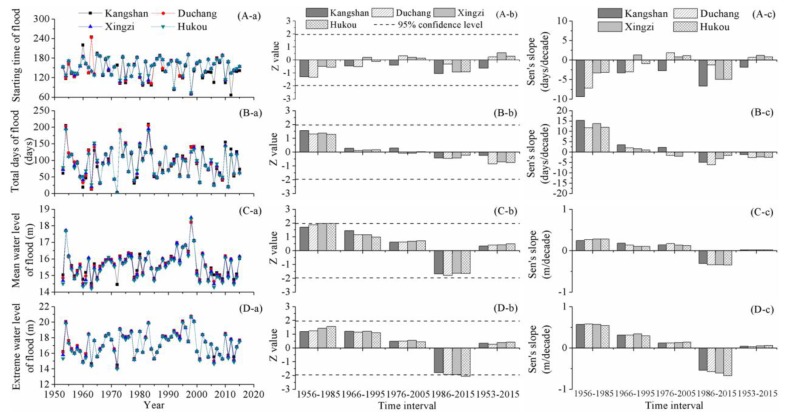
Change features of flood in Poyang Lake: (i) the sub-figures labeled (A–D) display change features of starting time of flood, total days of flood, mean water level of floods, and extreme water level of floods, respectively; and (ii) the sub-figures labeled (a–c) display annual values, Z values, and Sen’s slopes, respectively.

Figure 5 depicts the spatial and temporal variations in flood features. The starting time of flood remained steady, which fluctuated with a small Sen’s slope and no significant trend was detected during 1953–2015. Likewise, at the short-term time scale, there was no significant alteration that could be identified by the M-K algorithm. For the total days of flood, no prominent tendency was detected, at the short-term or long-term time scale. In summary, the starting time and duration of flood changed slightly.

Overall, the mean water level and extreme water level of floods experienced analogical change patterns. During the periods of 1956–1985, 1966–1995, and 1976–2005, the water level of floods was dominated by an increasing tendency. From 1986 onwards, a downward trend was detected. During the time interval of 1986–2015, the downward trend was not significant for the mean water level of floods, but was important for the extreme water level of floods at Hukou station. Over the entire study phase, both the mean water level and extreme water level of floods remained relatively unchanged; the Sen’s slopes were small, no larger than 0.1 m/decade. A catastrophic flood with the flooded area up to 239,000 km^2^ occurred during July to August in 1998. During 1953–2015, this flood was the largest one, followed by the flood in 1954. Relatively high mean and extreme water levels of these two floods could be observed obviously in Figure 5. The highest occurrence rates of severe floods were identified to be during the 1990s, when the flood-affected agricultural areas reached the highest levels. The abnormally large inflows from the local sub-basins and subsequent large streamflow of Yangtze River were mainly responsible for the severe floods in 1990s. Furthermore, the smallest storage capacity of Poyang Lake in 1990s due to floodplain occupancy and levee construction increased the severity of floods [59].

### 4.5. Drought Characteristics

**Figure 6:**
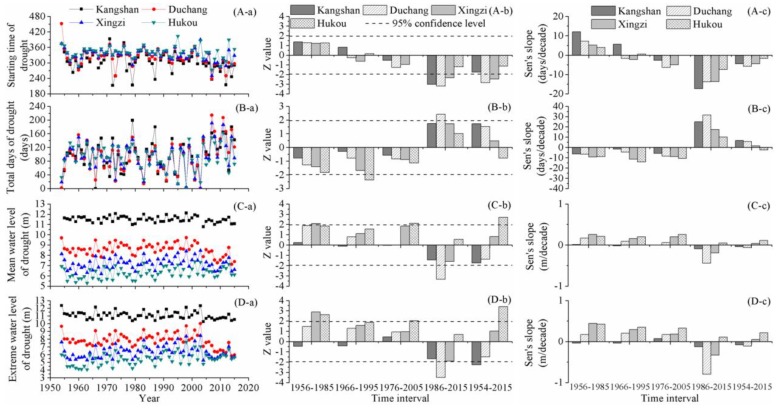
Change characteristics of drought in Poyang Lake: (i) the sub-figures labeled (A–D) display change features of starting time of droughts, total days of drought, mean water level of droughts, and extreme water level of droughts, respectively; and (ii) the sub-figures labeled (a–c) display annual values, Z values, and Sen’s slopes, respectively.

Spatial and temporal variations in drought characteristics are displayed in Figure 6. From 1956 to 2005, the starting time of drought remained steady and no prominent alteration was detected during the short-term time intervals. However, over the past 30 years, the downward trend was dominant at the starting time of droughts, indicating that Poyang Lake entered the dry season earlier than before. Over the long-term time interval, the downward trend was significant at the beginning of droughts at Duchang and Xingzi stations. Notably, no prominent alteration was detected at Hukou station.

Before 2005, there were consistent decreasing tendencies in the drought duration, but the significant alteration was sparse. Only a significant downward trend was identified at Hukou station over the period of 1966–1995. The total days of drought increased during 1986–2015 and the trend passed the significance test of 95% confidence level at Duchang station. The results indicated that the drought duration was prolonged in recent years. Although the drought duration fluctuated at the short-term time scale, there was no significant trend at the long-term time scale.

At Kangshan station, the mean water level of droughts changed slightly and the M-K algorithm did not detect a significant trend at different time intervals. At Duchang station, a prominent decreasing trend was identified during the time interval of 1986–2015. At Xingzi station, the mean water level of droughts increased persistently until 2005, while declined slightly from 1986 to 2015. At Hukou station, the upward tendency of the mean water level of droughts was dominant. It was notable that over the entire study phase, the mean water level of droughts was elevated significantly at Hukou station, while it dropped slightly at Kangshan and Duchang stations.

Over the entire study phase, the spatial pattern of changes in extreme water level of droughts was analogous to that of changes in mean water level of droughts. The extreme water level of droughts declined significantly at Kangshan station, while it increased prominently at Hukou station. At the short-term time scale, the change patterns were dominated by the increasing tendencies over the periods of 1956–1985, 1966–1995 and 1976–2005. However, during 1986–2015, the downward tendency was dominant, particularly at Duchang station, which monitored a significant decreasing trend.

### 4.6. Rate and Frequency of Water Level Changes

**Figure 7:**
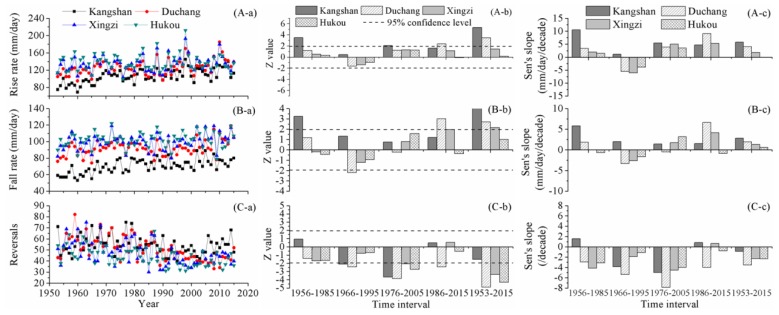
Variation features of fluctuating rates and reversals of water level in Poyang Lake: (i) the sub-figures labeled (A–C) display change features of rise rate, fall rate, and reversals, respectively; and (ii) the sub-figures labeled (a–c) display annual values, Z values, and Sen’s slopes, respectively.

Figure 7 illustrates the spatial and temporal variations in rate and frequency of water level changes. At Kangshan station, the rise rate of water level increased to some extent in different time intervals. Additionally, the significance of the upward trends passed the M-K test of 95% confidence level during the time intervals of 1956–1985, 1976–2005 and 1953–2015. At Duchang station, over the periods of 1956–1985, 1966–1995 and 1976–2005, the rise rate changed slightly, indicated by small Z values, which were within the threshold ranges of 5% significance level. However, during the time intervals of 1986–2015 and 1953–2015, the rise rate increased markedly at Duchang station. At Xingzi and Hukou stations, the rise rate varied slightly and no prominent trend was detected at different time intervals. Particularly at Hukou station, the Sen’s slope over 1953–2015 was small, almost equal to 0 mm/day/10a.

The change features of fall rate were similar to that of rise rate. At Kangshan station, the upward trends were dominant at different time intervals. The fall rate increased prominently during the periods of 1956–1985 and 1953–2015. At Duchang station, the fall rate fluctuated to different extent during different time intervals: increased slightly during 1953–1985, decreased significantly during 1966–1995, declined slightly during 1976–2005, and increased prominently during 1986–2015 and 1953–2015. At Xingzi station, significant upward trends were detected during the time intervals of 1986–2015 and 1953–2015. At Hukou station, there was no significant upward or downward trend detected in the fall rate at different time intervals.

Overall, the temporal variation features of reversals were dominated by downward tendencies and sparse upward tendencies were detected. During the time interval of 1956–1985, no significant trend was detected in the reversals of water levels at the four hydrometric stations. The reversals of water levels at Kangshan and Duchang stations decreased prominently during 1966–1995, and 10 years later, the reversals of water levels at the four hydrometric stations declined significantly during 1976–2005. Over the entire study phase, the downward trends were dominant and the reversals of water levels decreased significantly at Duchang, Xingzi, and Hukou stations.

## 5. Discussion

### 5.1. Implication for Environmental Problems

Trend analysis of the 47 water level indicators indicated distinguishing change features at long-term and short-term time scales. There were varied temporal and spatial change characteristics in different aspects of lake water regime. The significance and magnitude of the trend varied for different water level indicators over different time intervals and at different positions of Poyang Lake. It was worth noting that over the entire study phase of 1953 to 2015, the monthly mean water level in October, the monthly minimum water level in October, and the monthly minimum water level in November declined significantly and consistently at Kangshan, Duchang, Xingzi, and Hukou stations. The water level reduction was particularly dramatic in recent 30 years. The change slope of the water level drop in October was the largest among the change slopes of the monthly water levels. The monthly mean water level in November also decreased prominently at Duchang, Xingzi, and Hukou stations. For the rest water level indicators, no common significant upward or downward tendencies could be detected at the four hydrometric stations in Poyang Lake. The results indicated that the most dramatically altered indicators were the monthly water levels in October and November, which is the retreating season of Poyang Lake. Therefore, an urgent environmental problem that Poyang Lake faced was the water level decline and water resources reduction in October and November, which might impair the wetlands and lacustrine ecosystems.

A potential environmental crisis that Poyang Lake faced was the prolonged periods of drought. Although over the period of 1953 to 2015, no significant tendency was detected in the time series of total days of drought, the drought period increased over the past three decades, especially at Duchang station, which monitored a significant increase in drought period. The water level reduction in October and November triggered the drought earlier than normal, and resulted in the prolonged drought period. Significant downward trends have been identified at the beginning of droughts at Duchang and Xingzi stations over the past three and six decades. If no strategy was applied to mitigate the water level decline during the retreating season, a systematic and significant change could be expected in the starting time and duration of droughts in Poyang Lake.

### 5.2. Potential Ecological Impact

Hydrological features, which affect many functions of wetlands around Poyang Lake, have been recognized as major driving forces for vegetation growth, distribution, and composition. Different vegetation communities react differently to water level fluctuations. For instance, certain communities, e.g., *Carex* and *Eremochloa ophiuroides*, are capable to survive a wide variety of mean water depth and percent time inundated, while others, like *Carex-Polygonum criopolitanum*, are found to be relatively sensitive to hydrological conditions [60].

An investigation in Poyang Lake National Nature Reserve during the retreating period of 2001–2010 indicated that annual mean biomass of vegetation had significantly positive correlation with the exposure days of lake bottom, verifying that the water level was a key factor of biomass variations [61]. Based on this investigation, it could be speculated that water level reduction during October and November, increasing the drought duration and the exposure days of lake bottom, might have some positive impact on vegetation biomass. The altered inundation pattern caused by water level decline led to an increase in the mudflat area that is suitable for the growth of vegetation [62]. As a result, the vegetation area increased and typically spread toward the lake center [63]. During 1973 to 2013, the vegetation coverage has experienced a statistically significant increase with a rate of 15.9 km^2^/year [64].

However, the increased vegetation coverage and biomass was a result of the recession of wetlands, which turned to be dry lands due to water level decline. It should be noted that the decrease in water level during the retreating season had a seriously negative impact on ecological succession of wetlands around Poyang Lake, which is one of the world’s six major wetland systems and serves as a unique and important ecosystem [65]. The degraded wetland ecosystem directly affected the habitat and survival of migratory wintering birds, including the highly endangered Siberian crane (*Grus leucogeranus*). Almost 95% of the entire population of the Siberian crane wintered in the Poyang Lake region, where they foraged on the tubers of the submerged aquatic macrophyte *Vallisneria spiralis* [66]. As a result of the early drying of wetlands in October and November, some of the native aquatic vegetation communities risked to be replaced by upland vegetation, putting the wintering water birds in danger due to the shortage of food. The increase in wetland exposure would seriously deteriorate the wetland system and threaten the endangered migratory wintering birds that inhabit it. In Poyang Lake region, the ecological values of maintaining wetland system and providing suitable habitat for wintering birds are much greater than the ecological values of the increased vegetation coverage. Therefore, it is necessary to implement effective countermeasures to mitigate the water level decline and wetland recession.

### 5.3. Possible Causing Factors

**Figure 8:**
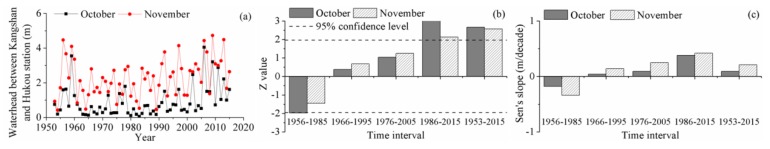
Change features of waterhead between Kangshan and Hukou station: the sub-figures labeled (a–c) display annual values, Z values, and Sen’s slopes, respectively.

The water budget of Poyang Lake is affected mainly by the basin meteorological conditions (e.g., precipitation and evapotranspiration), volume of runoff that is discharged into lake, volume of water that pours out of lake, and socio-economic water consumptions. The hydraulic gradient from southern to northern lake is an important factor affecting the drainage of Poyang Lake. A larger hydraulic gradient indicates a weakened storage capacity and an enhanced drainage capacity, and finally resulted in the decrease in water level and water resources of Poyang Lake. In order to investigate the change features of hydraulic gradients during October and November, the waterhead between southern and northern Poyang Lake was calculated using the monthly mean water level at Kangshan station minus the monthly mean water level at Hukou station. The trend analysis was then conducted; the results are shown in Figure 8. The hydraulic gradient between southern and northern lake equals the waterhead between Kangshan and Hukou station divided by the distance between the two stations. Therefore, the change features of waterhead reflected the change features of the hydraulic gradient.

Overall, there were analogous change features of the waterhead in October and November. At the long-term time scale of six decades, the time series of waterhead experienced a significant upward trend. At the short-term time scale of three decades, the time series of waterhead remained relatively steady and no prominent change could be detected during the time intervals of 1966 to 1995 and 1976 to 2005. However, a dramatic increase of waterhead emerged from 1986 to 2015. The results indicated that the hydraulic gradient between southern and northern Poyang Lake in October and November has increased significantly, particularly over the recent 30 years. Therefore, the drop of water level in October and November could be partially attributed to the enhanced discharge capability of Poyang Lake.

### 5.4. Recommendations for Environmental Managers and Future Works

The analysis results of Sen’s slopes indicated that the water level in October and November declined more dramatically at Hukou station than at Kangshan station, resulting in the increased waterhead between southern and northern Poyang Lake. The water level at Hukou station is highly correlated with the water level of the mainstream of Yangtze River. An increased water level of Yangtze River would block Poyang Lake, reduce its hydraulic gradient, and restrict its pouring. Eventually, the water level in Poyang Lake was expected to be elevated, and the recession of wetland ecosystem would be avoided. Therefore, a proper water level of Yangtze River was suggested to maintain the reasonable water level of Poyang Lake, which could be named environmental water level for the protection of lacustrine and wetland ecosystems. The attendant issue needed to be addressed is how much the environmental water level of Poyang Lake is. For effective conservation of wetland and wintering birds, more efforts are necessary to analyze: (a) the impact of changing water regime on vegetation communities and key food plants of the birds; and (b) the optimal management of lake to ensure the availability of adequate habitat. The impact of water regime alterations needs to be further assessed based on long-term monitoring of the wetland ecosystem, covering a wide range of parameters related to hydrological features, water quality, vegetation and associated fauna.

The optimal management of Poyang Lake partially relies on the management of Yangtze River, the hydrological processes of which affect the water regime of Poyang Lake through “blocking effect” and “emptying effect” [26]. Given an environmental water level of Poyang Lake, the environmental water requirement of Yangtze River needs to be assessed to sustain the natural river-lake interactions. In Yangtze River, the environmental water requirements of fish spawning and water quality of estuary have been studied [67,68]. Further effort is necessary to mitigate the altered river-lake interactions. The environmental flow assessment of a river’s blocking effect on a lake in a river-lake system may be helpful to address this issue [69].

The alterations in water regime of Poyang Lake are caused by the interplay of climate change and anthropogenic activities, although the two causal factors may have different contributions. Using the long-term monitored water level data, the present study investigated the spatial-temporal variations in water regime and pointed out the urgent environmental problem. However, due to limitation of raw data on precipitation, temperature, evapotranspiration, sand mining, reservoir regulation, and socio-economic water consumption, the present study failed to reveal the driving mechanisms behind the water regime alterations. Future efforts should be made to quantify the contribution of each impact factor, and the results will help policy makers to design scientific management strategies.

### 5.5. Direction of the Water Level Indicators

The proposed 47 water level indicators were applied in Poyang Lake. Combined with the trend analysis technologies, the spatio-temporal variations were studied and the results indicated the urgent environmental problem and help to design the coping strategy. The application shows the reliability and practicability of the indicators. When applying the water level indicators in other international lakes, the series of indicators can be used in whole or in part. If there is no clear hypothesis, the study can simply analyze every possible water level indicator and detect which comes out as most variable. If the study concerns a specific environmental issue, only several indicators need to be analyzed. For example, in a specific lake, if it has been known that the flood is a key factor affecting the lacustrine ecosystem, the indicators characterizing the flood are necessary and the other indicators are dispensable. Which indictor needs to be used is determined mainly by the ecohydrological features of a lake and the research objectives.

## 6. Conclusions

The water regime of large seasonal lakes is characterized by the hydrological features of magnitude, timing, duration, change rage and frequency. The proposed 47 indicators can quantify the hydrological features and be categorized into four groups, including (a) magnitude of monthly water level; (b) timing, duration, and magnitude of flood; (c) timing, duration, and magnitude of drought; and (d) rise rate, fall rate, and frequency of water level changes. Combined with the technologies of trend detection, the indicators are reliable and effective to indicate the evolution in lake water regime. The application in Poyang Lake shows the practicability of the indicators, which can be used in other international seasonal lakes. The selection of indicators is determined mainly by the local interest, e.g., the ecohydrological characters of the specific lake and the research objectives.

As a case study, the water regime of Poyang Lake has experienced some significant changes due to ongoing climate change and intensive anthropogenic activities. The rounded analysis of temporal and spatial alterations in the water regime of Poyang Lake drew the following conclusions:

(a) Over the long-term time scale, the flood regime remained relatively steady and no significant trend could be detected in the time series of starting time, duration, mean water level, and extreme water level. In the months from June to September during the high water level season, there was no prominent tendency in the time series of monthly mean water level, monthly maximum water level, and monthly minimum water level. At the short-term time scale, sparse significant alterations could be detected in the time series about flood characteristics. In the past 30 years, the mean water level and extreme water level of floods were dominated by downward trends. The spatial variation of flood regime changes was not obvious.

(b) The drought regime has experienced some significant changes during the past six decades and the spatial variation was obvious. At the long-term time scale, the mean water level and extreme water level of droughts in the southern Poyang Lake decreased, while the water levels in the northern lake increased. At the short-term time scale, the time series of mean and extreme water levels were dominated by increasing tendency before 2005. In the past 30 years, the time series of mean and extreme water levels decreased, particularly at the middle Poyang Lake. A notable change in drought regime was the decreasing trend in the time series of starting time of drought. In the last six decades, especially the last three, a significant downward trend was detected in the time series of starting time of drought, indicating that the drought occurred earlier. The concomitant alteration was the prolonged duration of drought from 1986 to 2015.

(c) The rise rate and fall rate of water levels experienced similar changes. During the time intervals at the short-term time scale, sparse significant increasing and decreasing trends could be detected. At the long-term time scale, the time series of rise rate and fall rate were dominated by an upward tendency, and the magnitude of change diminished in a longitudinal direction from south to north. At both long-term and short-term time scales, the number of water level reversals mainly decreased.

(d) For monthly water levels, the water regime experienced the most dramatic changes in October, followed by November. The noticeable alteration was the dramatic drop of water levels over the entire study phase, particularly in the recent 30 years. The drop of water level could be partially ascribed to the increased hydraulic gradient between the southern and northern lake, which enhanced the drainage capability of Poyang Lake. The water level reduction could trigger the drought occurring earlier, prolong the drought duration, and eventually place immense pressure on the wetland ecosystem, which provides habitat for the migratory wintering birds. In order to resolve this urgent environmental problem that Poyang Lake faced, the natural river-lake interactions and environmental water requirements of both Poyang Lake and Yangtze River need to be further investigated in future works.

## Figures and Tables

**Table 1:** Water level indicators.

Group	Number of Hydrologic Parameters	Hydrologic Parameter	Unit
Magnitude of monthly water level	36	Mean water level in each month	m
		Maximum water level in each month	m
		Minimum water level in each month	m
Timing, duration, and magnitude of flood	4	Starting time of flood	-
		Total days of flood	days
		Mean water level of floods	m
		Extreme water level of floods	m
Timing, duration, and magnitude of drought	4	Starting time of drought	-
		Total days of drought	days
		Mean water level of droughts	m
		Extreme water level of droughts	m
Rate and frequency of water level changes	3	Rise rate	mm/day
		Fall rate	mm/day
		Number of water level reversals	-
